# Editorial: Brain aging, neurodegeneration, and the role of natural molecules in maintaining brain health

**DOI:** 10.3389/fnins.2025.1717873

**Published:** 2025-11-12

**Authors:** Małgorzata Kujawska, Grzegorz Kreiner, Pierre-Louis Teissedre

**Affiliations:** 1 Department of Toxicology, Poznan University of Medical Sciences, Poznan, Poland; 2 Maj Institute of Pharmacology, Polish Academy of Sciences, Kraków, Poland; 3 Université de Bordeaux, Institut des Sciences de la Vigne et du Vin, UMR 1366 Œnologie, Villenave d' Ornon, France

**Keywords:** brain aging, neurodegeneration, natural molecules, proteostasis, neuroinflammation, precision medicine, translational strategies

The demographic shift toward an aging global population has made brain health one of the most pressing scientific and public health challenges of our time. Neurodegenerative diseases such as Alzheimer's and Parkinson's impose an increasing burden on individuals and societies, while effective preventive or disease-modifying therapies remain elusive (Sabayan et al., 2023). This gap has fueled interest in natural molecules and nature-inspired derivatives as agents capable of reinforcing endogenous defense mechanisms, modulating proteostasis, and reducing neuroinflammation (Kujawska and Jodynis-Liebert, 2018; Babazadeh et al., 2023). By integrating advances in genetics, mechanistic neuroscience, and translational strategies, researchers are beginning to build a more coherent framework for preserving brain function across the lifespan (Nehmeh et al., 2024; Jiang et al., 2025).

A first theme concerns the link between inherited genetic variation and the vulnerability of cortical networks. Using a multimodal imaging-genetics approach, Kim et al. identified single nucleotide polymorphisms associated with cortical thinning and cognitive decline and showed that amyloid-β burden partially mediates these effects. The work highlights the convergence of immune-synaptic signaling pathways and cortical atrophy, underscoring the importance of stratifying patient cohorts not only by clinical phenotype but also by genotype and amyloid status (Kim et al.). Beyond methodological innovation, these findings indicate that genetic information may serve as an important factor to consider in the design of preventive or interventional trials, supporting therapeutic strategies that are better aligned with the biological context of vulnerability (Kim et al.; Nehmeh et al., 2024). Together, these findings suggest that molecular interventions may ultimately need to be guided by precision medicine principles, where genomic risk scores and amyloid imaging inform both trial inclusion and expected responsiveness.

Mechanistic insights into neuronal survival emerge in complementary fashion from the work of Wu et al., who disrupted the Sel1L-Hrd1 ER-associated degradation (ERAD) complex selectively in adult neurons. Loss of ERAD function destabilizes endoplasmic reticulum homeostasis, triggers maladaptive unfolded protein responses, and leads to progressive hippocampal and cerebellar degeneration with behavioral manifestations of ataxia (Wu et al.). These findings establish ER proteostasis not merely as a biomarker of disease but as a prerequisite for neuronal viability.

Building on this mechanistic foundation, Kouchmeshky et al. introduced ellorarxine, a selective retinoic acid receptor agonist with favorable stability compared to all-trans retinoic acid. In neuronal cultures, ellorarxine mitigated excitotoxic injury, restored proteostasis, and modulated stress granule dynamics. While the data are preclinical, they illustrate how molecules inspired by endogenous signaling can reinforce resilience under conditions of synaptic stress and proteostatic challenge. Importantly, the study highlights the translational potential of receptor-specific retinoids, which may overcome the pharmacokinetic and safety limitations of earlier compounds (Kouchmeshky et al.). This line of work also connects to a broader literature indicating that retinoid signaling intersects with pathways of neuroinflammation and plasticity, making it a promising yet still underexplored therapeutic avenue (Mey and Mccaffery, 2004; Kouchmeshky et al.).

A complementary translational avenue is offered by repurposed iron chelators. Fine et al. tested intranasal deferoxamine in the intracerebroventricular streptozotocin model of sporadic Alzheimer's disease. Low-dose treatment reversed deficits in spatial memory, prevented hippocampal neuronal loss, and attenuated neuroinflammatory and degenerative signatures at both the transcriptomic and cytokine levels. Higher doses were less effective, emphasizing the need for careful dose selection. The use of intranasal delivery to bypass the blood–brain barrier is particularly notable, as it opens a practical route for chronic administration of chelators and other molecules with limited systemic tolerability (Fine et al.).

Taken together, these studies not only establish ER proteostasis as a prerequisite for neuronal survival but also provide a mechanistic support for therapeutic approaches aimed at sustaining proteostasis, suggesting that interventions targeting stress granules, protein clearance, or retinoid pathways may derive part of their efficacy from stabilizing ER function. At the same time, rigorous dose-finding and long-term evaluation will be essential to define the therapeutic window and safety profile of such agents (Jiang et al., 2025).

The contributions in this Research Topic outline a coherent translational arc. Genetic risk factors set the stage for vulnerability; proteostatic networks in the endoplasmic reticulum determine neuronal survival, and nature-inspired interventions, whether receptor-selective retinoids or repurposed chelators, offer strategies to restore resilience.

The convergence of these dimensions underscores the need for interdisciplinary research that bridges scales—from genomic risk to molecular targets to functional outcomes. Equally critical will be the development of regulatory frameworks capable of accommodating nutraceuticals, repurposed drugs, and synthetic derivatives of natural molecules, ensuring that promising leads can be tested in well-designed clinical trials (Kakoti et al., 2022; Qiao et al., 2023; Feldman et al., 2024; Jiang et al., 2025).

In conclusion, the articles collected here demonstrate how complementary approaches can be integrated into a forward-looking strategy for preserving brain health across aging. By linking genetics, mechanistic insight, and translational intervention, this Research Topic emphasizes the value of sustained collaboration across disciplines and sectors. Future progress is likely to require genetics-informed patient selection, robust mechanistic endpoints, and pragmatic delivery routes that together can accelerate the safe and effective deployment of natural molecules and their derivatives to protect the aging brain.
